# The Trp-rich Antimicrobial Amphiphiles With Intramolecular Aromatic Interactions for the Treatment of Bacterial Infection

**DOI:** 10.3389/fmicb.2021.733441

**Published:** 2021-10-04

**Authors:** Zhihua Wang, Qiuke Li, Jinze Li, Jiawei Li, Lu Shang, Shuli Chou, Yinfeng Lyu, Anshan Shan

**Affiliations:** The Laboratory of Molecular Nutrition and Immunity, Institute of Animal Nutrition, Northeast Agricultural University, Harbin, China

**Keywords:** antimicrobial peptide, intramolecular aromatic interaction, antimicrobial activity, bacterial infection, subcutaneous abscess

## Abstract

Antibiotic resistance is emerging as a hot issue with the abuse and overuse of antibiotics, and the shortage of effective antimicrobial agents against multidrug resistant bacteria creates a huge problem to treat the threatening nosocomial skin and soft tissue infection. Antimicrobial peptides (AMPs) exhibite enormous potential as one of the most promising candidates of antibiotic to fight against pathogenic infections because of its unique membrane penetration mechanism to kill pathogens, whereas the clinical application of AMPs still faces the challenges of production cost, stability, safety, and design strategy. Herein, a series of Trp-rich peptides was designed following the principle of paired Trp plated at the *i*th and *i*th+4 position on the backbone of peptides, based on the template (VKKX)_4_, where X represents W, A, or L, to study the effect of intramolecular aromatic interactions on the bioactivity of AMPs. Through comparing the antimicrobial performance, hemolysis, cytotoxicity, and stability, VW5 which is equipped with the characters of direct antimicrobial efficacy (GM=1.68μM) and physical destruction of bacterial membrane (SEM and electron microscopy) stood out from the engineering peptides. VW5 also performed well in mice models, which could significantly decrease the bacterial colony (VW5 vs infection group, 12.72±2.26 vs 5.52±2.01×10^9^CFU/abscess), the area of dermo-necrosis (VW5 vs infection group, 0.74±0.29 vs 1.86±0.98mm^2^) and the inflammation cytokine levels at the abscess site without causing toxicity to the skin. Overall, this study provides a strategy and template to diminish the randomness in the exploration and design of novel peptides.

## Introduction

The cutaneous abscess, formed from the accumulated pus and located underneath the dermis, is one of the most common symptoms of skin and soft tissue infection induced by the invasion of pyogenic bacteria (pus producing bacteria) with the sign of redness, swelling, warmth, and pain (Brook and Frazier, 1997). There is no need to carry out medical intervention for the abscess, which is associated with transient painful lumps; however, a combination of surgically drained and antibiotic treatment is necessary to treat the severe cases of large deep abscess with the complication of bloodstream dissemination, traditionally (Trowbridge, 2010). While the therapeutic efficiency of antibiotics was weakened once the abscess formed and excessed to a certain size, there are complex components (the debris of necrotic cells, fibrin, bacteria, and so on) and abnormal pathological change (decreased pH level and low redox potential) at the site of the abscess (Bryant and Mazza, 1989). In addition, the antibiotic resistant bacteria with high virulent was continuously emerging along with the misuse and overuse of antibiotics for the past decades. For example, the well-known ESKAPE pathogens were the most recalcitrant resistant bacteria causing hospital acquired infection (*Enterococcus faecium*, *Staphylococcus aureus*, *Klebsiella pneumoniae*, *Acinetobacter baumannii*, *Pseudomonas aeruginosa,* and *Enterobacter species*; Pletzer et al., 2017; Su et al., 2020). Therefore, the shortage of efficient antimicrobial agents was an urgent problem to be solved to cope with various resistant bacterial infections (Xue et al., 2021).

Antimicrobial peptides (AMPs) have been elected as one of the most potential alternatives of antibiotics to deal with the increasing shortage of antimicrobial agents fighting against antibiotic resistant bacteria because of the distinguished mode of action (Dong et al., 2014a; Song et al., 2020). The physical membrane permeabilization depends on the well-known amphipathicity of AMPs, which could be interpreted as the characters of electro-positivity and hydrophobicity amino acid residues located at different sides on the backbone of peptides (Lyu et al., 2019). The cationic residues on the polar face of peptide generated the identification ability of negative bacterial membranes through electrostatic interaction and the hydrophobic residues on other side were mainly provided the insertion force for the peptide to disrupt the bacterial membrane (Tossi et al., 2000). Whereas the clinical application of most natural AMPs still faced the challenge of complicated physiological environment, undesired cost, and potential systemic toxicity (Di et al., 2020). Therefore, numerous single-factor modification strategies have been developed to enhance the structural features and functional diversity of AMPs, including the truncation of sequence, substitution of residues, hybridization of functional sequence, cyclization of the peptides, and so on (Wang et al., 2019; Elliott et al., 2020).

It was well discussed that the hydrogen bond of an α-helical structure, formed by the backbone N-H group (*i*th) and the backbone C=O group (*i*th+4), was the essential factor to stabilize the peptide secondary structure (Lyu et al., 2019). Beside the hydrophilic interaction of the hydrogen bond on the backbone, the interactions between the side chain of residues also played a vital role to maintain the low-energy structure, structural stability, and diversity of bioactivity of the peptide (Bi et al., 2013). The intramolecular aromatic interactions (π–π interactions of benzene rings) were exactly the representative side chain interactions which could impact the structure and function of peptides among those side chain interactions (Waters, 2004). In addition, among the aromatic amino acids, tryptophan was frequently employed to design a novel peptide because of the unique bulky indole ring on the side chain which exhibited the affinity to near-surface of lipid and the headgroup of the membrane (Chan et al., 2006). However, there was not a lot of information and systematic research about the effect of intramolecular aromatic interactions on the bioactivity of AMPs. Therefore, a series of peptides was synthesized with the paired Trp residues located on the *i*th and *i*th+4 position on the backbone of the peptide using the template (VKKX)_4_, where X represents W, A, or L. Leu was introduced to the sequence to compare the effect of different types of hydrophobic side chain on the bioactivity of peptides (the paired aliphatic amino acid residues and the aromatic amino acid residues). The Ala residues are equipped with a non-bulky and chemically inert functional group, which was to help to mimic the structure preferences of other amino acids. Therefore, the Ala residues were employed to mutate specific Trp sites on the sequence to study the role of the specific site and the amounts of Trp-Trp pairs to the bioactivity of peptides (Sato et al., 2000). Then the antimicrobial activity, biocompatibility, the mode of action, and the *in vivo* antimicrobial activity of peptides was further studied.

## Materials and Methods

### Strains and Peptides Synthesis

The tested strains were kindly provided by the laboratory of Veterinary Microbiology and Immunology, the College of Veterinary Medicine, Northeast Agricultural University (gram-negative bacteria, *Salmonella enterica serovar typhimurium* C77-31, *Salmonella typhimurium* ATCC 14028, *Escherichia coli* ATCC 25922, *Pseudomonas aeruginosa* ATCC 27853 and gram-positive bacteria, *Staphylococcus aureus* ATCC 29213, *Staphylococcus aureus* ATCC 43300, *Staphylococcus epidermidis* ATCC 12228, *Staphylococcus aureus* ATCC 25923).

The well-known solid-phase method was employed to synthesize the testing peptides (GL Biochem, Shanghai, China) and the C-terminal of peptides was amidated with the purpose of the stabilization of the secondary structure and the supply of the extra positive (Liu et al., 2008). In addition, the synthesized peptides were purified by RP-HPLC to reach the >95% purity at 220nm with a nonlinear water/acetonitrile gradient (contained 0.1% trifluoroacetic acid) under the flow rate of 1.0ml/min. Then, the peptides were further characterized by MALDI-TOF MS (Linear Scientific Inc., U.S.) and analytical RP-HPLC (GS-120-5-C18-BIO 4.6×250mm column). The power of synthesized peptides was dissolved in DI water for further testing.

### CD Spectrum

The circular dichroism (CD) spectrum was further determined by analysis the secondary structure of peptides in different membrane-mimic environments (Dong et al., 2014b). The two totally different mimic environments were 10mM PBS and 50% TFE solution and the final concentration of peptides was fixed at 150μM Then the samples were transferred into a rectangular quartz cuvette (1mm path length) and further underwent the scanning of J-820 spectropolarimeter (Jasco, Tokyo, Japan) at the speed of 50nm/min. The wavelengths ranged from 190 to 250nm and were recorded to plot the observed ellipticity curve. The observed value at each time point was the average of three scans. The results were presented of mean residue ellipticity, which was calculated from the observed ellipticity as follows.

$$\theta_{M}=\left(\theta_{\mathrm{obs}}\cdot 1000\right)/(c\cdot l\cdot n);$$

(Zhu et al., 2014a)

The meaning of each letter was displayed as followed: θ_M_, residue ellipticity [deg.cm^2^.dmol^−1^]; θ_obs_, observed ellipticity [mdeg]; c, peptide concentration [mM]; l, path length [mm]; n, the number of amino acid residues of peptide.

### Hemolytic Activity

The hemolytic activity of peptides was reflected by the releasing amount of hemoglobin from erythrocytes lysis induced by peptides (Dong et al., 2014a). The informed consent was obtained from the volunteers (3 healthy 25-year-old male volunteers) before the experiment. In brief, the human red blood cells (hRBCs), donated by volunteer, were collected, washed, and resuspended in PBS at pH 7.2. The peptides were serially diluted in PBS at different concentrations in a 96-well cell cultures plate and incubated with 50μl hRBCs for 1h at 37°C. The 0.1% Trition X-100 was selected as the treatment of positive control and the negative control was the hRBCs treated with PBS. After 1h incubation, the supernatant, obtained by centrifuging the plate 1,000g for 5min, was transferred to a brand-new plate for absorbance detection at 570nm. The releasing amount of hemoglobin was converted to the percent hemolysis against to 0.1% Triton X-100 treatment. The lowest concentration caused 5% hemolysis of hRBCs and was defined as the lowest hemolytic concentration of antimicrobial peptide.

### Cytotoxicity

The MTT (3-[4,5 dimethylthiozol-2-yl]-2,5-diphenylterazoliumbromide) dye was selected to analyze the cytotoxicity of peptides against the murine macrophage cell RAW 264.7 (National Collection of Authenticated Cell Cultures, Shanghai, China), human embryonic kidneys (HK293T), and the porcine intestinal epithelial cells (IPEC-J2) as in our previously study (Li et al., 2020). In short, the cells (10^4^ cells/well) were seeded into 96-well plates and were cultivated overnight. Then the peptides with different final concentration (2μM, 4μM, 8μM, 16μM, 32μM, 64μM, and 128μM) were introduced into the plate and challenged with cells for 24h. After overnight incubation, the 50μl MTT (0.5mg/ml) was added into the cell cultures for 4h. Then the plate was centrifuged to discard the supernatants and obtain the formazan crystals. The DMSO (dimethyl sulfoxide) was employed as a solvent to dissolve the formazan crystals on the bottom of the plate and the optical density was read at 492nm with a microplate reader.

### Antimicrobial Activity

The famous microtiter dilution, National Committee for Clinical Laboratory Standards, was adopted to measure the antimicrobial ability of the engineering peptides (Chou et al., 2016). In brief, the 50μl with serial dilution was incubated with the same volume bacteria (1×10^5^CFU/ml in midi-logarithmic phase) in a 96-well plate 18–24h at 37°C. The positive control was the bacteria treated with culture medium and the negative control was merely culture medium. The MICs (minimum inhibitory concentrations) were defined as the lowest concentration of peptides that prevented the production of visible turbidity (Tossi et al., 2000).

### Time-Kill Curve and Fractional Survival Tests

The logarithmic phase bacterial cells (*P. aeruginosa* ATCC 27853 and *S. aureus* ATCC 29213) were harvested and diluted to 1×10^5^CFU/ml (Dou et al., 2017). The testing peptides at different concentrations (1×MIC, 2×MIC, and 4×MIC) were challenged with bacteria at a series of time intervals (0, 5, 10, 30, 60, and 120min). Then the reaction system was diluted several times and plated on Mueller-Hinton agar (MHA, AoBoX, Shanghai, China) plates. The plates were incubated 24h at 37°C in order to count the bacterial colonies. The data were the means of three times independent assays.

### Salt and Serum Sensitivity Assays

The antimicrobial performance of peptides against *P. aeruginosa* 27853 cells was further studied under the complicated physiological conditions (Lyu et al., 2016). The concentrations of salts were selected as the same as the physiological concentration to test the effect of antimicrobial activity (150mM NaCl, 4.5mM KCl, 6μM NH_4_Cl, 8μM ZnCl_2_, 1mM MgCl_2_, and 4μM FeCl_3_). The serum was also taken into the consideration for the adverse impact on the antimicrobial effect of peptides. The AMPs were operated at different concentrations of human heat-inactivated serum (12.5, 25, and 50%) as previously studied (Ma et al., 2014).

### LPS Binding

The BODIPY-TR-cadaverine (BC) displacement assay was performed to analyze the LPS binding ability as previously studied (Ma et al., 2012). At the beginning, the LPS-probe solution was formed by 4h incubation of 50μg/ml LPS (derived from *Pseudomonas aeruginosa*, purchased from Sigma-Aldrich, Shanghai, China) and 5μg/mlBC dye in Tris buffer. After which, the peptides with different concentrations (1μM, 2μM, 4μM, 8μM, 16μM, 32μM, and 64μM) were added into a mixture to react for 1h and the fluorescence values were detected (excitation λ=580nm, emission λ=620nm). The result of LPS-binding ability of peptides was calculated as the percentage against fluorescence value induced by 10μg/ml Polymyxin B.

### Outer Membrane Permeabilization

The outer membrane permeabilization of peptides was observed by monitoring the fluorescence change of dye 1-N-phenylnaphthylamine (NPN; Dong et al., 2014c). The 10μm NPN (Sigma-Aldrich, Shanghai, China) was first incubated with the *P. aeruginosa* ATCC 27853 cells (OD_600_=0.2, suspended in 5mM HEPES buffer supplied with 5mM glucose) for 30min. Afterward, the mixture was subjected to the detection of fluorescence, which served as the background at the excitation wavelength (*λ*=350nm) and the emission wavelength (*λ*=420nm). Then the peptides with various concentrations (1μM, 2μM, 4μM, 8μM, 16μM, 32μM, and 64μM) were added into the mixture and the fluorescence was monitored until the value was tended to stable. The %NPN uptake against 10μg/ml polymyxin B was calculated to analyze the outer membrane permeability of peptides.

### Cytoplasmic Membrane Electrical Potential

The cytoplasmic membrane depolarization of peptides was analyzed with membrane potential-sensitive dye DISC_3_-5 (Ma et al., 2011). First, 0.4μM DISC_3_-5 (Sigma-Aldrich, Shanghai, China) was incubated with the *P. aeruginosa* ATCC 27853 cells (OD_600_=0.05) suspended in 5mM HEPES buffer supplied with 20mM glucose for 1h. Then 100mMK^+^ was introduced into the mixture for the reason of keeping K^+^ concentration equilibration of bacterial cytoplasmic and extracellular conditions. Afterward, the peptides were reacted with the bacterial cells and the fluorescence value was tracked over time to plot the fluorescence curve (excitation λ=622nm, emission λ=670nm).

### Scanning Electron Microscope

The field emission scanning electron microscope (SEM) was adopted to analyze the morphology changes of the bacterial membrane as previously studied (Dong et al., 2012a). In short, the bacterial cells (*P. aeruginosa* ATCC 27853 and *S. aureus* ATCC 29213) in the exponential growth phase were harvested and treated with the peptides at MIC values for 30min. Then, the bacterial cells were fixed with glutaraldehyde (2.5% w/v) overnight and dehydrated with various concentration ethanol solutions (50, 70, 90, and 100%) 10 times. Subsequently, the bacterial cells were further incubated with a mixture containing half volume of alcohol and half volume of tert-butanol 30min and pure tert-butanol for 1h. The critical point dryer was employed to dry the bacterial cells and the SEM was introduced to visualize the morphology.

### Transmission Electron Microscope

The sample for the test of the Transmission Electron Microscope (TEM) was to follow the same procedure as SEM. After the overnight pre-fixation with 2.5% glutaraldehyde, the bacteria was then post-fixed with 2% osmium tetroxide (sealed ampule, Shanghai, China). Afterward, the samples were dehydrated with ethanol at a series of concentrations (50, 70, 90, and 100%) and substituted the 100% ethanol with 50% ethanol+50% acetone, 100% acetone, 50% acetone+50% epoxy resin, and 100% epoxy resin successively. Finally, the samples were sectioned and stained for observation.

### Subcutaneous Abscess Model

The *in vivo* antimicrobial performance of peptides was assessed by the treatment effect of the subcutaneous bacterial abscess model (Pletzer et al., 2017). In brief, 24 female ICR mice (weighted 18–20g, WeiTonglihua Co., Ltd. (Beijing)) were divided into four groups and each group contained 6 mice (*n*=6). The back hair of mice was carefully cleaned, shaved, and sterilized for the preparation of the experiment. Then, the *Pseudomonas aeruginosa* ATCC 27853 cells (100μl, 10^8^CFU/ml) were injected into the subcutaneous layer of the dorsum skin. 1hour after infection, the mice were treated with saline, VW5 (5mg/kg), and ciprofloxacin (5mg/kg) intraperitoneally and the treatments were repeated twice a day until the experiment was finished. The non-infected mice served as a control, which was infected and treated with saline.

### Assessment of Bacterial Infection

The therapeutic effect of peptides was further evaluated by colony counting, and the appearance and the level of inflammatory cytokine (Tan et al., 2020). The appearance of abscesses was taking photos and the area of dermonecrotic tissue was measured and calculated with calipers at Day 3. The infected skin on the back of the mouse was harvested entirely and homogenized with saline at Day 3. The homogenate of abscess was spread on the MHA plates and cultivated for 18–24h at 37°C. Then the bacterial colony was counted for data analysis. The serum was collected for proinflammatory cytokines (TNF-*α*, IL-1α, IL-1β, and IL-2) tests at Day 3. The cytokine levels were determined by commercial ELISA kits (Nanjing Jiancheng Bioengineering Institute, Nanjing, China).

### Histological Examination

The histopathology of abscesses lesions was further analyzed by hematoxylin/eosin (HE) staining (Aladdin, Shanghai, China). The tissue samples at Day 3 were excised and fixed in 4% paraformaldehyde solution. In addition, the cell nuclei were stained blue and the cytoplasm was stained pink.

### Skin Toxicity

The potential toxicity of peptides to normal skin tissues was further tested by TUNEL assay (Huang et al., 2009). The back hair of mice was carefully removed and was injected with 100μl 100% DMSO, saline, and VW5 (5mg/kg) subcutaneously for 24h. The 100μl 100% DMSO treatment served as a positive control, which was well-known for its toxicity to induce skin necrosis. Then, the skin samples were harvested and were subjected to TUNEL assay and counterstain with 4,6-diamidino-2-phenylindole (DAPI).

### Statistical Method

The data were derived from the three times independent experiments and are presented as means ± standard deviation with the analysis of ANOVA by SPSS 16.0 software. In addition, the statistical significance involved in this work was regarded as value of *p*<0.05.

## Result

### Characterization of the Peptides

**Figure 1:**
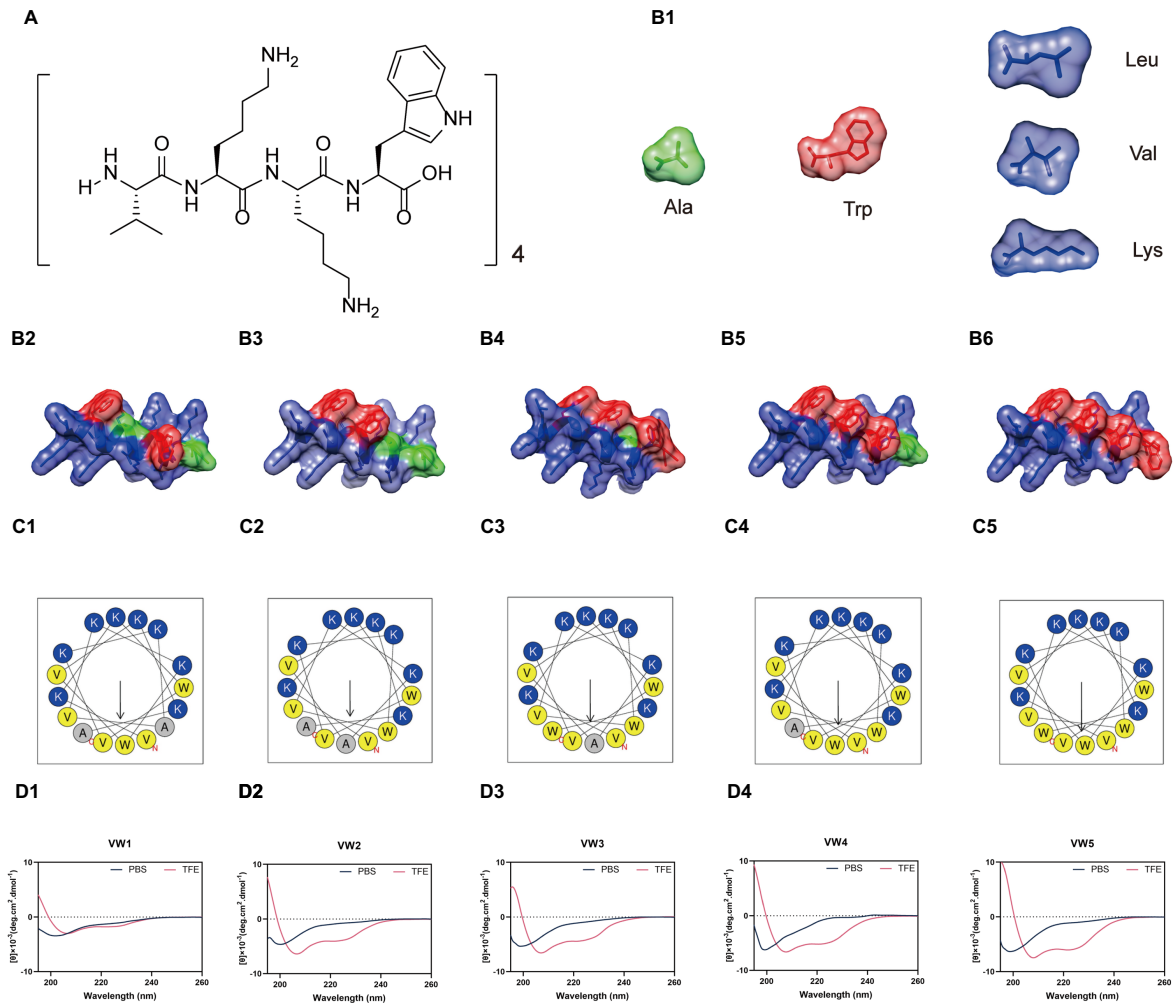
**(A)** Schematic structure of peptides based on the template (VKKX)_4_, where X represents W, A, or L. **(B)** The backbone structure and potential surface of peptides. The green structure represents Ala residues, the red structure is Trp residues, and the blue structure represents other amino acid residues involved in the sequence. **(C)** The helical wheel projections of peptides. The charged amino acid residues are colored as dark blue and the hydrophobic amino acid residues are painted as dark yellow. **(D)** The circular dichroism (CD) spectrum of peptides.

It was suggested by the consistency of theoretical molecular weights and measured molecular weights that the peptides were successfully synthesized and purified (confirmed by MALDI-TOF MOS, matrix-assisted laser desorption/ionization time-of-fight mass spectrometry; Table 1). The engineering peptides were designed following the template (VKKX)_4_, where X represents W, A, or L, and the paired Trp was decreased from 3 to 0 (VW5 to VW1) accompanying the Ala involved into the sequence to mute the Trp residues (Figures 1A,B). The values of measured and theoretical molecular weights were the same, which was indicated in that all peptides were successful synthesized. The net charge of all engineering peptides was maintained at +9 and the peptides exhibited amphipathic structure from the helical wheel (Figure 1C). As the paired Trp residues were introduced to the sequence, the hydrophobicity was gradually increased from 0.13 to 0.37 (Table 1). Even the VW1 and VW2 exhibited the same amino acid composition and hydrophobicity, they are not equipped with the same amount of paired Trp residues after the substitution of Ala residues (VW1 equipped with 0 paired Trp residues and VW2 equipped with 1 paired Trp residues). The same condition also occurred in VW3 and VW4, which showed 1 and 2 paired Trp residues, respectively. Among those peptides, VW5 equipped with 3 paired Trp residues, which was the most amount of paired Trp residues.

**Table 1:** Peptide design and key physicochemical parameters.

Peptide	Sequence	Formula	TMW^a^	MMW^b^	Net charge	H^c^
VL1	VKK*L*VKK*A*VKK*L*VKK*A*-NH_2_	C_86_H_166_N_24_O_17_	1808.41	1808.57	+9	0.06
VL2	VKK*L*VKK*L*VKK*A*VKK*A*-NH_2_	C_86_H_166_N_24_O_17_	1808.41	1808.59	+9	0.06
VL3	VKK*L*VKKLVKK*L*VKK*A*-NH_2_	C_89_H_172_N_24_O_17_	1850.50	1850.61	+9	0.15
VL4	VKK*L*VKK*L*VKK*A*VKK*L*-NH_2_	C_89_H_172_N_24_O_17_	1850.50	1850.64	+9	0.15
VL5	VKK*L*VKK*L*VKK*L*VKK*L*-NH_2_	C_92_H_178_N_24_O_17_	1892.58	1892.72	+9	0.24
VW1	VKK*W*VKK*A*VKK*W*VKK*A*-NH_2_	C_96_H_164_N_26_O_17_	1954.52	1954.60	+9	0.13
VW2	VKK*W*VKK*W*VKK*A*VKK*A*-NH_2_	C_96_H_164_N_26_O_17_	1954.52	1954.62	+9	0.13
VW3	VKK*W*VKK*W*VKK*A*VKK*W*-NH_2_	C_104_H_169_N_27_O_17_	2069.66	2069.76	+9	0.25
VW4	VKK*W*VKK*W*VKK*W*VKK*A*-NH_2_	C_104_H_169_N_27_O_17_	2069.66	2069.73	+9	0.25
VW5	VKK*W*VKK*W*VKK*W*VKK*W*-NH_2_	C_112_H_174_N_28_O_17_	2184.79	2184.90	+9	0.37
Melittin	GIGAVLKVLTTGLPALISWIKRKRQQ-NH_2_	C_131_H_229_N_39_O_31_	2846.47	2846.00	+6	−0.033

a *TMW, theoretical molecular weight*.

b *MMW, measured molecular weight*.

c *H, the mean hydrophobicity of the peptide, which was analyzed by the HeliQuest (http://heliquest.ipmc.cnrs.fr/cgi-bin/ComputParamsV2.py)*.

### CD Spectrum of Peptides

The secondary structure of peptides in different membranes mimics the environment, which was determined by CD spectroscopy (the aqueous environment which was mimicked by 10mM sodium phosphate buffer and the hydrophobic environment which was mimicked by 50% TFE). As shown in Figure 1D, the peptide VW5 (150μM) exhibited unordered conformation in the sodium phosphate buffer. Whereas the α-helical tendency could be detected with the spectrum character of two negative peaks at about 208 and 222nm when the peptide existed in the 50% TFE.

### Antimicrobial Activity

The MIC was employed to measure the antimicrobial activity of peptides against a panel of gram-negative and gram-positive bacteria (Tables 2, 3). In general, the broad-spectrum antimicrobial activity of most of the peptides could be observed at the test concentration. The antimicrobial activity of Trp substituted derivatives equipped with relative higher antimicrobial activity compared to its Leu substituted derivative counterparts. The antimicrobial activity of engineering peptides was gradually increased as the amount of paired Trp or Leu occurring in the sequence. The geometric mean (GM) of the MIC value was also calculated to evaluate the relative antimicrobial performance of engineering peptides (the GM of VW1, VW2, VW3, VW4, and VW5 was 76.11μM, 41.50μM, 7.34μM, 3.36μM, and 1.68μM). Among those peptides, VW5 exhibited outstanding antimicrobial activity compared to other peptides and equipped with the same GM (1.68) as the well-known membrane disruption peptide melittin (GM=1.68μM). In addition, the peptide VW5 showed effective antimicrobial activity against *P. aeruginosa* ATCC 27853 with the value of MIC of 1μM.

**Table 2:** MICs of AMPs (μM).

MIC^a^	VL1	VL2	VL3	VL4	VL5	VW1	VW2	VW3	VW4	VW5	ME^b^
**Gram-negative**											
*S.typhimurium* 7731	>64	>64	32	16	2	>64	64	8	2	1	1
*S.typhimurium* 14028	>64	>64	32	32	4	64	64	8	4	2	1
*E. coli* 25922	>64	>64	16	16	8	64	8	4	4	2	1
*P.aeruginosa* 27853	>64	64	8	8	2	32	8	4	2	1	2
**Gram-positive**											
*S.aureus* 29213	>64	>64	16	16	8	64	64	16	4	2	4
*S.epidermidis* 12228	>64	>64	32	32	4	64	64	8	4	2	1
*S.aureus* 25923	>64	>64	32	16	8	>64	64	8	4	2	2
MRSA 43300	>64	>64	32	32	8	>64	64	8	4	2	4

a *MICs, minimum inhibitory concentration*.

b *ME, melittin*.

**Table 3:** The biocompatibility of antimicrobial peptides.

Peptides	GM_MIC_^a^ (μM)	HC_5_^b^ (μM)	IC_50_^c^ (μM)			Therapeutc index (TI)^d^				GM_TI_^e^
		hRBC	IPEC-J2	HEK 293T	RAW264.7	hRBC	IPEC-J2	HEK 293T	RAW264.7	
VL1	128.00	>128	7,508	9,615	8,795	>1.00	58.65	75.40	68.71	>23.48
VL2	117.38	>128	10,301	8,411	7,516	>1.09	87.76	71.65	64.03	>25.74
VL3	22.63	>128	8,955	8,217	6,258	>5.66	395.72	363.11	276.55	>122.44
VL4	19.03	>128	4,045	7,278	4,147	>6.73	212.53	382.42	217.91	>104.47
VL5	4.76	>128	4,559	7,494	1924	>26.89	957.83	1574.26	404.20	>357.80
VW1	76.11	>128	9,192	6,608	6,691	>1.68	120.77	86.82	87.91	>35.28
VW2	41.50	>128	5,663	5,978	5,665	>3.08	136.45	144.05	136.50	>53.63
VW3	7.34	>128	4,749	10,639	4,750	>17.44	646.97	1449.42	647.10	>320.73
VW4	3.36	>128	6,236	9,246	6,235	>38.10	1855.95	2751.71	1855.65	>775.15
VW5	1.68	>128	5,126	8,551	3,126	>76.19	3051.34	5090.03	1860.86	>1218.17
ME	1.68	1	3.34	5.38	3.79	0.59	1.99	3.18	2.26	1.71

a GM_MIC_ means the geometric mean of the MICs.

b HC_5_, MHC is the lowest concentration of peptide that caused 5% hemolysis of human red blood cells.

c IC_50_ was the half maximal inhibitory concentration.

d TI, therapeutic index, is the ratio of MHC to GM or IC_50_ to GM, which was reflecting the cell selectivity of peptides.

e GM_TI_ represents the geometric mean of TI of various cell lines.

### Hemolytic Activity and the Cell Selectivity

**Figure 2:**
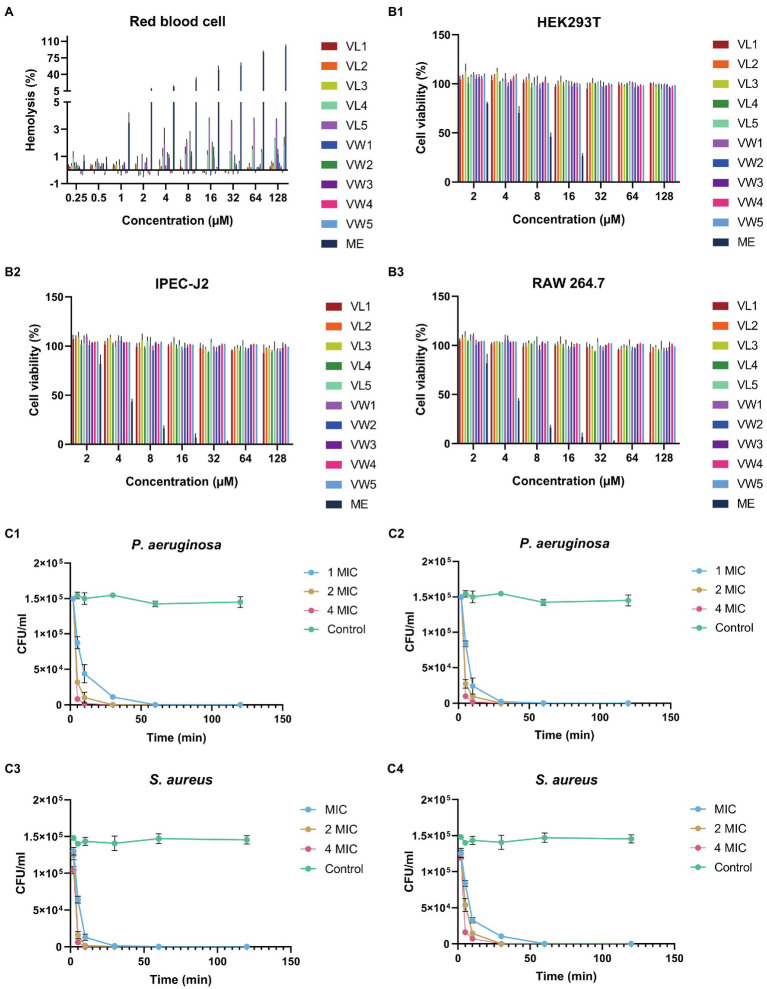
**(A)** The hemolysis activity of peptides at different concentration (0.25μM, 0.5μM, 1μM, 2μM, 4μM, 8μM, 16μM, 32μM, 64μM, and 128μM). **(B)** Cytotoxicity of engineering peptide to HEK293T **(B1)**, IPEC-J2 **(B2)**, and RAW 264.7 cells **(B3)** followed by the MTT methods at different concentrations (2μM, 4μM, 8μM, 16μM, 32μM, 64μM, and 128μM). The data represented as means ± standard deviations of three independent experiments. **(C)** The killing curve of peptides against bacteria. The *eruginosa* ATCC 27853 and *S. aureus* ATCC 29213 were diluted into1×10^5^CFU/ml, and treated with VW5 **(C1,C3)** and melittin **(C3, C4)** at their 1×MIC, 2×MIC and 4×MIC after 0, 5, 10, 30, 60, and 120min treatment. The colony counting was performed to determine the survival rate of bacterial cells by plating on MHA plates.

To further research the potential toxicity to mammalian cells of peptides, which was hindered by the clinical application, the hemolytic activity of peptides against hRBCs was further determined. Among those peptides, all peptides maintained excellent biocompatibility which did not cause 5% hemolytic activities even at the highest test concentration of 128μM. The well-known AMPs melittin exhibited terrible hemolytic activity which could cause 5% hRBCs hemolysis at 1μM (Figure 2A and Table 3).

### Cytotoxicity

To measure the toxicity of peptides to eukaryotic cells, the MTT methods were employed to detect the cell viability of RAW 264.7, HEK 293T, and IPEC J2 after the treatments of peptides at a series of concentrations (Figure 2B). The result of cytotoxicity exhibited similar results as the hemolytic activity and the whole series of peptides did not exhibite cytotoxicity to those three types of cells even at the highest test concentration. The cell viability of VW5 was maintained at about 91.29% (RAW 264.7) and 97.93% (IPEC J2) at the highest test concentration of 128μM. When the cytotoxicity of the positive control melittin was tested, the cell viability was significantly decreased compared to engineering peptides and the cell viability was below 5% at 32μM (both RAW 264.7 and PEC J2).

### Cell Selectivity

The cell selectivity of peptides to bacteria over mammalian cells was reflected by the calculation of the therapeutic index (TI, the ratio of MHC to GM or IC_50_ to GM), as shown in Table 3. It could be observed that the TI value in the environment of red blood cells was increased accompanying the paired Trp residues occurring in the sequence (TI of VW1, VW2, VW3, VW4, VW5 was >1.68, >3.08, >17.44, >38.1, and>76.19, successively). Among those peptides, VW5 displayed the stronger antimicrobial activity (GM was 1.68μM) and better biocompatibility (HC_5_ of hRBC was >128μM). In addition, the paired Trp substitution exhibited relative larger TI values compared to its aliphatic acid substituted counterparts, and the TI of VL5 was >26.89 which was about 2.8-fold smaller than VW5. Even though the antimicrobial activity of melittin was excellent, the higher hemolytic activity decreased its therapeutic index (TI of hRBC was 0.59 and GM was 1.71), which hindered its clinical application. Besides, the therapeutic index of peptide in the mammalian eukaryotic cell (RAW 264.7, HEK 293T, and IPEC J2) was also calculated to study the biocompatibility of peptides. Still, peptide F5 is equipped with the largest TI value in the environment of IPEC-J2 cells (3051.34), HEK 293T cells (5090.03), RAW 264.7 cells (1860.86), and the largest GM of TI (>1218.17).

### Salt and Serum Sensitivity

The stability of antimicrobial activity of AMPs could be influenced by the complex physiological environment, such as salts, serum, and so on. Thus, the antimicrobial performance of peptides against *P. aeruginosa* ATCC 27853 has been tested in the presence of various salts and serum (Table 4). The most potential candidate VW5 exhibited relative stability antimicrobial activity (1μM) in the presence of most salts (Na^+^, K^+^, NH_4_^+^, Zn^2+^, and Fe^3+^) excepts for the divalent cation (Ca^2+^ and Mg^2+^). The MIC values of VW5 were evaluated twofold (2μM) against *P. aeruginosa* ATCC 27853 in the presence of Ca^2+^ and Mg^2+^ at their physiological concentration. The MIC value of F5 did not change compared to the control in the presence of 12.5% serum, but the MIC value increased twofold (2μM) in 25% serum and fourfold in 50% serum.

**Table 4:** MICs (μM) of peptides against *P. aeruginosa* in the presence of salts^a^ and serum.

	VL1	VL2	VL3	VL4	VL5	VW1	VW2	VW3	VW4	VW5
Control	>64	64	8	8	2	32	8	4	2	1
NaCl	>64	>64	32	8	4	64	16	8	2	1
KCL	>64	>64	16	16	4	32	8	4	2	1
NH_4_Cl	>64	>64	16	8	2	32	8	4	2	1
MgCl_2_	>64	>64	32	8	2	32	16	8	4	2
CaCl_2_	>64	>64	32	16	4	>64	8	8	4	2
ZnCl_2_	>64	>64	16	8	4	32	8	4	2	1
FeCl_3_	>64	>64	16	8	2	32	8	4	2	1
12.5% serum	>64	>64	16	8	4	32	8	4	2	1
25% serum	>64	>64	32	16	4	64	16	8	2	2
50% serum	>64	>64	64	32	16	>64	64	16	8	4

a *The concentration of salts was fixed at their physiological concentration (150mM NaCl, 4.5mM KCl, 6 μM NH_4_Cl, 1mM MgCl_2_, 2.5μM CaCl_2_, 8μM ZnCl_2_, and 4μM FeCl_3_), and the MIC values of the control were measured in the absence of these salts and serum*.

### Time Killing Curve

According to the performance of antimicrobial activity and toxicity, VW5 emerged as the most outstanding candidate among those peptides. Therefore, the killing effectives of VW5 was determined by treating *P. aeruginosa* ATCC 27853 with 1×MIC, 2×MIC, and 4×MIC as a function of time (Figure 2C). It could be observed that the killing effect of VW5 followed the manner of concentration-and time-dependence. The number of bacterial cells decreased rapidly after the treatment of VW5, and the bacterial cells were eradicated completely by 1×MIC (1μM) of VW5 in the system in 30min (10min and 30min were taken by 2×MIC (2μM) and 4×MIC (4μM) of VW5, respectively). The melittin exhibited the same trends, which could effectively kill the bacterial cells in 30min at their MIC concentration.

### LPS Binding Assay

**Figure 3:**
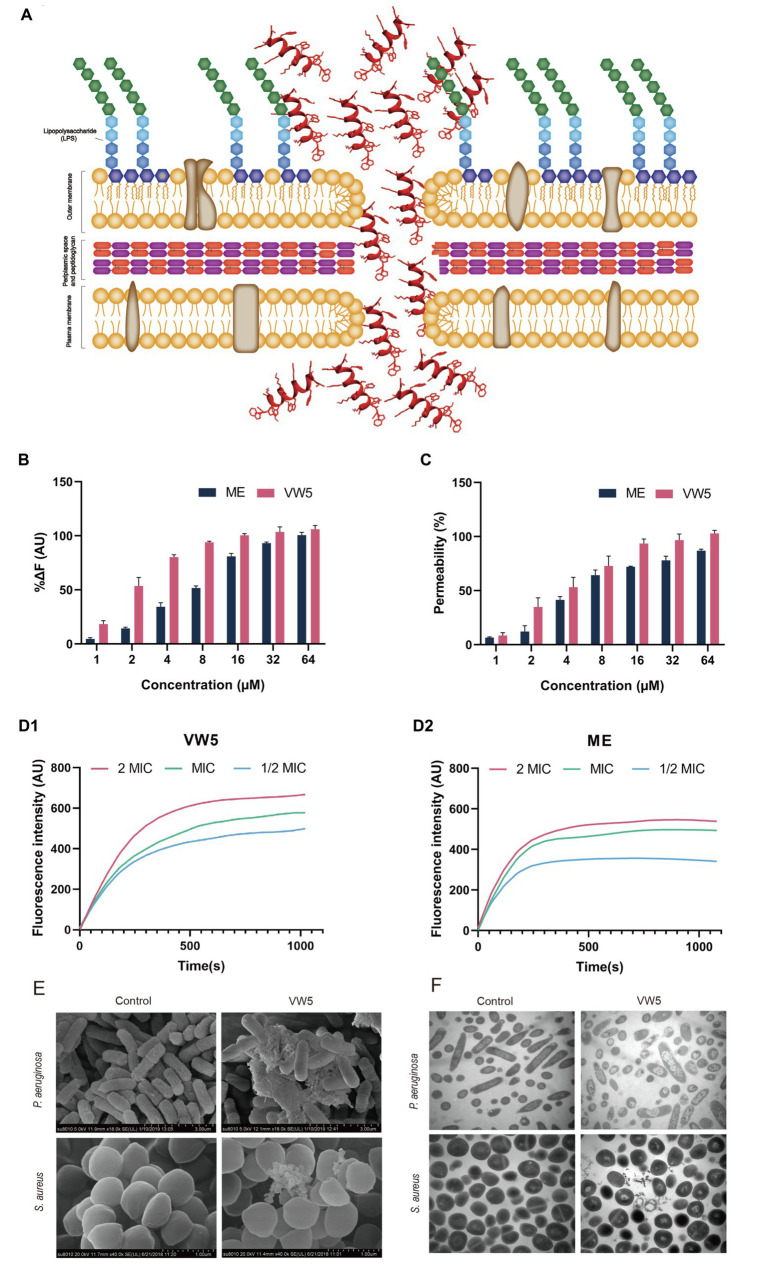
**(A)** Schematic of the model of action of antimicrobial peptide. **(B)** LPS binding affinity of VW5 at different concentrations. The BODIPY-TR cadaverine fluorescent dye displacement assay was employed to evaluate the LPS binding activity of peptides at an excitation wavelength of 580nm and an emission wavelength of 620nm. **(C)** The outer membrane permeabilization of *P. aeruginosa* ATCC 27853 cells. The fluorescence change of 1-N-phenylnaphthylamine (NPN) dye was used to study the outer membrane permeabilization after the treatment of VW5 and melittin at a series of concentrations (1μM, 2μM, 4μM, 8μM, 16μM, 32μM, and 64μM). **(D)** Cytoplasmic membrane depolarization of *P. aeruginosa* ATCC 27853 after incubation of VW5 and melittin at 2 MIC, MIC, and 1/2 MIC concentration. **(E)** Scanning electron microscopic micrographs of *P. aeruginosa* ATCC 27853 and *S. aureus* ATCC 29213 after the treatment of VW5 at MIC (1μM for *P. aeruginosa* ATCC 27853 and 2μM for *S. aureus* ATCC 29213). **(F)** Transmission electron microscope of bacterial cells treated with VW5. The bacterial specimen of control received no treatment.

To fully study the antimicrobial mechanism of antimicrobial peptide to fight against bacteria, the ability of peptide to bind lipopolysaccharide (LPS from *P. aeruginosa*) was determined using the well-known BODIPY-TR-cadaverine dye displacement method (Figures 3A,B). It could be observed that there was a dose-dependent manner occurring in the binding activity of VW5 to LPS, in which the fluorescence was gradually enhanced from 18.37 to 106.10%, which was compared with 10μg/ml polymyxin B accompanying the increase of the concentration of peptide from 1 to 64μM (the binding activity for melittin was raised from 4.59 to 100.57%). When compared with fluorescence reaction after the melittin treatment, VW5 exhibited relative higher LPS binding ability than melittin, especially in the low concentration 1 to 8μm (VW5 vs. melittin, 18.37% vs. 4.59% at 1μM, 53.56% vs. 14.28% at 2μM, 80.28% vs. 34.33% at 4μM and 93.91% vs. 51.67% at 8μM).

### Outer Membrane Permeability

The ability of VW5 to penetrate through the outer membrane, the first barrier of gram-negative bacteria to protect itself from the invasion of antibiotic, was determined in *P. aeruginosa* with the fluorescent dye NPN (N-phenyl-1-naphthylamine). In order to accurately evaluate the permeability of VW5, the well-known membrane-targeting antibiotics, polymyxin B, was selected as the standard and the fluorescence, which was induced by the 10μg/ml polymyxin B, was defined as 100% membrane penetration. With the increase of the concentration of VW5 and melittin (1 to 64μM), the fluorescent values were increasingly strengthened and the percentage ratio of peptide treatment to polymyxin B treatment continually rose from 9.49 to 102.91% after the treatment of VW5 and from 6.55 to 86.99% after the treatment of melittin at the concentration of 1 to 64μM. When considering the same concentration of VW5 and melittin, VW5 exhibited better permeability of the outer membrane than the control peptide melittin (Figure 3C).

### Cytoplasmic Membrane Depolarization

The potential-sensitive fluorescence dye diSC_3_-5 was employed to monitor the depolarization of cytoplasmic membrane of *P. aeruginosa* ATCC 27853 bacterial cells after the treatment of VW5 and melittin at their 1/2 MIC, MIC, and 2 MIC concentrations (0.5μM, 1μM, and 2μM of VW5 and 1μM, 2μM, and 4μM of melittin) and analyzed the disturbance of the cytoplasmic membrane potential. The change of fluorescence after VW5 and melittin treatment over time was recorded and the curve of the increased fluorescence was corrected by subtracting the fluorescence of untreated bacterial cells which served as the background fluorescence. As the time past from 0s to 1,020s, the fluorescence reaction of VW5 was gradually increased until it reached its peak and maintained a relative stable status. In addition, the dose-dependent effect could also be observed, which was reflected by the fluorescence and was faster to reach its peak and kept at higher fluorescence levels at higher concentration compared to the same peptide at lower concentration (Figure 3D).

### Scanning Electron Microscope

The field emission scanning electron microscopy (FE-SEM) was employed to visualize the direct angle about the changes in surface morphology of *P. aeruginosa* ATCC 27853 and *S. aureus* ATCC 29213 (Figure 3E). The surface of untreated control *P. aeruginosa* ATCC 2785 was equipped with smooth and intact surfaces. After being treated with VW5 at MIC concentration (1μM), the bacterial surface of *P. aeruginosa* was corrugated, atrophied, and surrounded by a large amount of cell debris. A similar phenomenon also appeared in *S. aureus* ATCC 29213 cells after being treated with VW5 at MIC concentration (2μM). The *S. aureus* ATCC 29213 cells were equipped with smooth cell membranes and unbroken round cell structures. However, the surface of *S. aureus* ATCC 29213 cells was shrunken, and some cells were even broken into debris and discrete blebs.

### Transmission Electron Microscope

The TEM assay was also performed to revel the change in the ultrastructure of *P. aeruginosa* ATCC 27853 and *S. aureus* ATCC 29213 cells after the treatment of VW5. It could be observed in Figure 3F, the cytoplasm *P. aeruginosa* and *S. aureus* cell presented as the dense form, which was well protected by the intact bacterial membrane. When the VW5 at MIC concentration (1μM) was introduced into the reaction system, the cellular contents of bacterial cells leaked with the sign of the reduce intracellular contents and conspicuous cytoplasmic zones surrounding the empty cavity of *P. aeruginosa* ATCC 27853 cells. A similar phenomenon occurred in *S. aureus* ATCC 29213 after being treated with VW5 at MIC concentration (2μM). The structure of untreated *S. aureus* ATCC 29213 was intact, and the contents were well protected by unbroken cell membranes. Whereas the cytoplasm of *S. aureus* ATCC 29213 cells could be observed, which were diffused around the shrunken cells after treatment of VW5.

### The Therapeutic Effect of Peptides to Cutaneous Abscess

**Figure 4:**
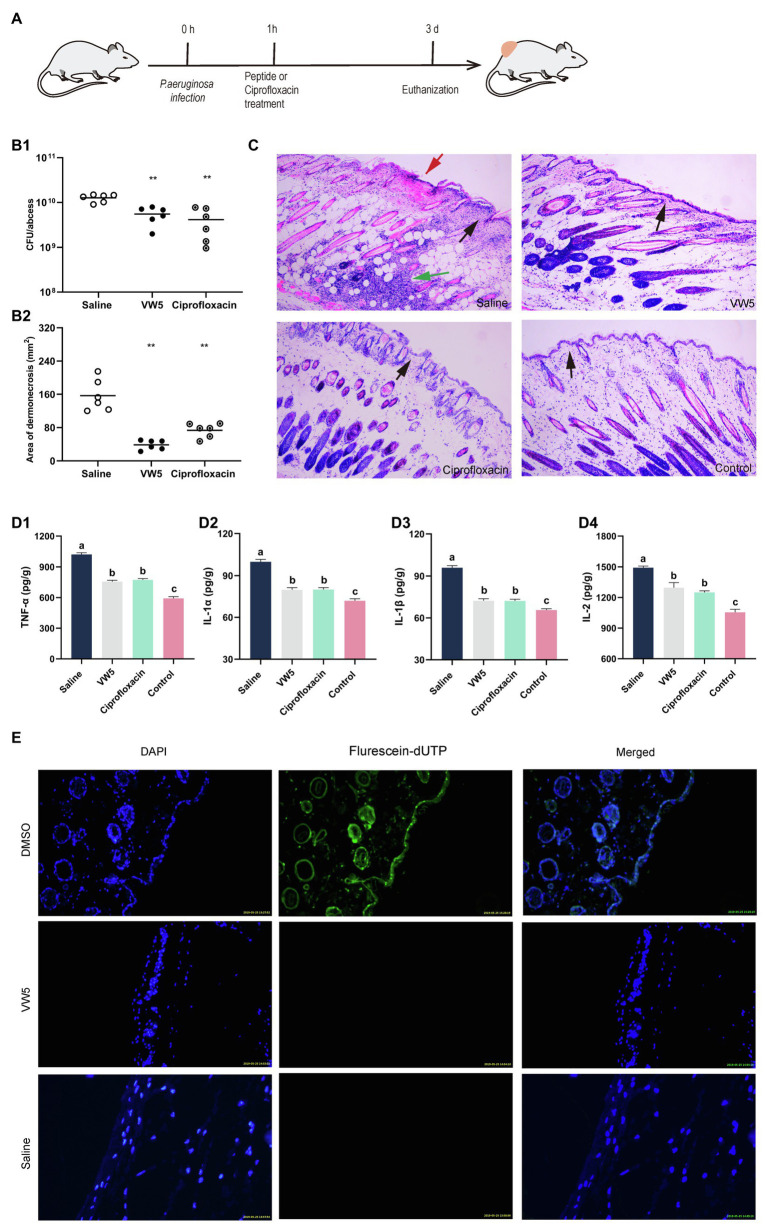
**(A)** Illustration of experimental protocol. **(B)** The therapeutic effect of peptides on bacterial abscess. The colony counting of abscess tissue **(B1)** and the area of lesion after treatment at 3 d. The saline group is the infected mice treated with equivalent saline and the control group refers to the uninfected mice. **(C)** The histological examination of abscess site by HE staining. The black arrows indicate the condition of the epidermis. The epidermis of the saline treatment was infiltrated with inflammatory cells, whereas the epidermis of VW5 treatment and ciprofloxacin treatment maintained integrity. Large-area necrosis tissue overlayed the epidermis (red arrow) of saline treatment at the abscess site. The cell debris and large amount of inflammatory cell infiltration could be observed in the dermis layer (green arrow). **(D)** The inflammatory cytokines level after the treatment of saline, VW5, and ciprofloxacin. **(E)** The skin toxicity of VW5. The TUNEL assay was performed to detect the apoptotic cells (green) in mice skin, and the nuclei of the cells were counterstained with DAPI (blue). DMSO (dimethyl sulfoxide) injection serves as a positive control.

The *in vivo* antimicrobial performance and therapeutic potential of VW5 were analyzed through the bacterial cutaneous abscess model. As shown in Figure 4, the scab (5mg/kg VW5 treatment and 5mg/kg ciprofloxacin treatment) was macroscopically reduced compared to the saline treatment and the area of abscess lesion was calculated to be VW5 (0.74±0.29) and ciprofloxacin (0.38±0.09), which was significantly decreased compared to saline treatment (1.86±0.98). There was a significant difference of the bacterial load in the abscess lesion (VW5 vs. saline, 12.72±2.26 vs. 5.52±2.01×10 ^9^ CFU/abscess). In addition, the ciprofloxacin treatment and the VW5 treatment showed no significant difference in the scab area and the bacterial load (Figure 4B).

### Proinflammatory Cytokines Level

The proinflammatory cytokines (TNF-*α*, IL-1α, IL-1β, and IL-2) in serum were determined to estimate the therapeutic potential antimicrobial peptide VW5 (5mg/kg). According to Figure 4, the non-infection (control) maintained quite lower levels compared to the infected group no matter what kind of cytokines, in general (Figure 4D). Compared to saline treatment, VW5 treatment and ciprofloxacin treatment exhibited significantly reduced proinflammatory cytokine levels in serum (saline vs VW5 and ciprofloxacin: TNF-*α*, 1020.654±51.08 vs 756.62±39.12 and 773.35±40.55pg./ml; IL-1α, 99.95±4.71 vs 79.82±4.79 and 80.08±4.04pg./ml; IL-1β, 95.99±4.31 vs 72.36±4.15 and 72.18±3.54pg./ml; IL-2, 1492.65±48.11 vs 1296.25±150.86 and 1250.826±43.60pg./ml).

### Histological Examination

The histological examination was further applied to analyze the severity of inflammation in the abscess lesion and the integrity of skin structures. It is shown in Figure 4C that the structure of skin tissue was intact, which showed the epidermal layer, the dermal layer, and the subcutaneous adipose layers for healthy tissue. Several inflammatory responses occurred in the saline treatment with the character of extensive cell debris and inflammatory cells infiltration, and it also could be observed that the large-area necrosis tissue overlayed the epidermis at the abscess site. Nonetheless, the dermal tissue structures maintained relative integrity of the VW5 treatment (5mg/kg) and ciprofloxacin treatment (5mg/kg), and comparatively mild inflammatory response supported by less inflammatory cells aggregation in abscess lesions compared to the control skin, which was uninfected by bacteria.

### Toxicity of VW5 to Normal Skin Tissue

The TUNEL assay was performed to study the potential toxicity of peptide to normal skin tissue. The apoptotic cells in the epidermal layer were stained green with fluorescein dUTP and the cell nuclei was counterstained as blue by DAPI. The 100% DMSO was served as positive control because of the well-known cell toxicity. As shown in Figure 4E, the DMSO treatment was caused by heavy cell apoptosis and the epidermal cell was stained green with fluorescein-dUTP, which means obvious toxicity of skin tissue. The epidermal layer was stained with blue; however, no obvious green fluorescence occurred in the VW5 (5mg/kg) and saline treatment.

## Discussion

Because of the special membrane active action and antimicrobial performance, AMPs are increasingly becoming the hot topic at the field of antibiotic candidates in fighting against the emerging drug resistant bacterial infection (del Rosso et al., 2019; Zhang et al., 2020). Although the antimicrobial performance and research potency are promising, the clinical application of AMPs still faces great challenges such as manufacturing costs, safety, bioactivity, rational design strategy, and so on (Tan et al., 2021; Wang et al., 2021), which makes it important to explore the novel design strategy to design AMPs with high cell selectivity (Shao et al., 2020). It has been well discussed that the Trp residues played an important role in the antimicrobial activity of linear a-helical antimicrobial peptide by deeply inserting into the negatively charged bacterial membrane, and the penetration of AMPs into the bacterial membrane could be enhanced without causing obvious binding activity to the membrane of mammalian cells when the Trp residues occurred at the hydrophobic-hydrophilic interface (Song et al., 2019). This insertion of Trp-rich peptides was mainly dependent on the hydrogen bonds formed by aromatic side chain and the bilayer surface, which were equipped with a dipole moment of about 2.1D. Meanwhile, bacterial insertion activity of Trp-rich peptides mainly relied on the position of Trp residues rather than the amount of Trp residues occurring in the sequence (Chan et al., 2006; Shang et al., 2020). Therefore, the Trp residue was selected as the basic components for *de novo* design of AMPs in this study because of its superiority to bioactivity of peptide.

The aromatic interaction of side chains widely occurred at the same side of the helix structure in helical peptides and this noncovalent interaction played an important role in the structure of protein (Saravanan et al., 2014). The interaction between the aromatic rings at the *i* position and *i*+4 position in the sequence could minimize the energy landscape of peptide and provide the greatest stability through the interaction of intramolecular aromatic rings (Waters, 2004; Sanchez et al., 2011). Even in the role of the aromatic interaction of side chains on structure stability of peptides, there were few studies discussed about the relationship between the aromatic intramolecularly interactions and antimicrobial activity. The sequence of motif was the *de novo* design which was inspired by the strategy of activity units tandem (Ong et al., 2014). The general initial sequence was (XYYX)_n_, where the X is hydrophobic amino acid and Y is cationic amino acid, and n means the repeat units. In this sequence, the percentage of hydrophobic amino acid residues was maintained at 50% following the design principle as previously (Wang et al., 2016). As one of the most frequently charged amino acid residues occurred into the sequence of antimicrobial peptide according to the antimicrobial peptide database, the Lys residues were selected as the net positive charge donor to facilitate the targeting of cationic peptide to anionic bacterial membrane (Wang et al., 2016). The Val residues, which were usually used as elements of AMPs *de no* design, were employed to provide the hydrophobicity to promote the antimicrobial peptide to penetrate the hydrophobic core of the bacterial cell membrane (Tossi et al., 2000). Thus, VKKX was selected as the minimum activity unit and the repeat of sequence could work as the basic components to guarantee the generation of antimicrobial activity of the peptide base on previous studies (Ong et al., 2014).

Therefore, a series of Trp-rich peptides was synthesized with the paired Trp residues placed on the *i* position and *i*+4 position and the paired Leu residues were employed to substitute the Trp residues in the sequence compared to the effect of different types of amino acid on the antimicrobial activity. Finally, the specific site mutation of Ala residues was also studied to deplore the influence of the specific site of amino acid residues on the backbone and the number of Trp-Trp pairs occurred in the sequence on bioactivity of AMPs. According to the results of MICs, the antimicrobial activity of both series of peptides (Trp and Leu substitution) was gradually increased as hydrophobicity increased, which could promote the peptide to insert into the hydrophobicity core of the bacterial membrane (Lin et al., 2004). To deplore the amount of paired Trp residues on the antimicrobial activity, the Ala was introduced into the sequence to keep the hydrophobicity of peptides on the same condition and to eliminate the interference of hydrophobicity. When using the same template, the VW5 is equipped with stronger antimicrobial activity than VL5 compared to its Leu counterparts, which could be explained by the bulky indole ring side chain which could enhance the antimicrobial ability of peptides (Dong et al., 2014b). The antimicrobial activity of VW2, VW4, and VW5 gradually increased accompanied by more Trp residues introduced into the sequence, which could be explained by more Trp residues which could enhance the hydrophobicity of peptides and then strengthen the antimicrobial performance of the peptide (Dong et al., 2012b). Even the peptides VW3 and VW4 equipped with the same amino acid components and hydrophobicity, the VW 4, which has one more paired Trp residue, displayed obvious higher antimicrobial activity than VW3. In addition, the same condition also happened in the peptide VW1 and VW2, which indicates the antimicrobial activity could be improved by the increase of the amount of paired Trp residues when the hydrophobicity was maintained on the same condition. Meantime, it was also well reported that the position of Trp residues rather than the amount played an important role on the bioactivity of AMPs (Chan et al., 2006). Through comparing the helical wheel of VW3 and VW4, it could easily be found that the Trp residues of VW4 were introduced into the center of the hydrophobic face of the backbone but VW3 was into the noncentral site of the hydrophobic face. Even VW3 and VW4 equipped with the same amino acid composition, the VW4 possessed stronger amphipathicity, which has a profound influence on the biological activity of AMPs (Lyu et al., 2019).

The cell selectivity of the peptide was also investigated to analyze the clinical potential of Trp-rich peptides by the calculation of the therapeutic index, which was the ratio of MHC to MICs (Chou et al., 2016). Among those engineering peptides, all peptides did not show hemolysis and obvious toxicity to a eukaryotic cell, which could be explained by the moderate hydrophobicity of peptides. It has been reported that the moderate contents of hydrophobic residues would help to keep the hydrophobicity and the net charge on suitable balance (Zhu et al., 2014b). Even the peptide VW5, which has the highest hydrophobicity, did not show obvious cytotoxicity, which was firmly proved in the previous studies that the antimicrobial activity would be significantly increased without causing undesired hemolysis when the paired tryptophan was introduced into the sequence at the hydrogen bond position (Lyu et al., 2019). Among those peptides, VW5 stand out from the Trp derivatives because of the maximum TI, which means excellent cell selectivity.

The electrostatic attraction was the essential factor for peptides to identify and target a negative bacteria cell membrane; however, the complex physiological environment (mainly the cations and serum) was a huge challenge for AMPs to exert their antimicrobial activity by the electrostatic screening and binding effect (Fedders et al., 2008). The electrostatic screen effect has widely existed between the antimicrobial peptide and the positive charged cations (monovalent cations Na^+^, K^+^, and NH4^+^ and multivalent cations Ca^2+^, Mg^2+^ and Fe^3+^) as in previous studies (Chou et al., 2016; Dou et al., 2017). In addition, this well-known electrostatic screen effect could have a negative effect on the antimicrobial peptide to penetrate the bacterial membrane and further effect the antimicrobial performance of AMPs (Wang et al., 2018). In the current study, the antimicrobial activity of VW5 was kept stable in the presence of most of the physiological slats and was only slightly affected by Ca^2+^ and Mg^2+^, which suggested relative well stability. In addition, multivalent cations could also competitively bind to the anionic phosphate group of bacterial membrane and prompt the membrane to become more rigid, which would hinder the penetration effect. In the physiological environment, the plasma protein (mainly albumin) was another factor to weaken the antimicrobial effective to fight against various pathogens which could not be ignored. The MICs of candidate peptide VW5 were only increased fourfold in the 50% serum indicating its well performance in stability.

The interaction between AMPs and the bacterial membrane has been discussed for decades and the membrane permeabilization has been summarized as the main mechanism of AMPs to exert its antimicrobial activity (Hayouka et al., 2017). The negative charged components (mainly hydroxylated phospholipids and LPS) played a role as a target to attract positively charged AMPs toward the bacterial membrane by electrostatic attraction (Zhang et al., 2019b; Dong et al., 2020; Fiorentino et al., 2021). The LPS binding ability of VW5 was carried out by fluorescence-based displacement assay, and the efficiency of LPS binding of VW5 exhibited higher than melittin, which was known for its strong LPS binding ability. The concentration was gradually increased with gathering peptides to prepare the transmembrane action on a local site until the threshold was broken, and then the peptides obtained the initial force to penetrate the membrane and in the meantime the conformation of peptides was changed due to the change from an aqueous to a hydrophobic environment (Giangaspero et al., 2001; Zhang et al., 2019a). Thus, the peptides could further insert into the cytoplasmic membrane inducing a series of chain reactions included the disturbance of membrane electrical potential, the destruction of membrane integrity, the leakage of contents, and the death of bacteria ultimately (Forde et al., 2018). Based on those theories, the effect of AMPs on the cytoplasmic membrane and inner membrane were further studied using NPN and diSC_3_-5. The result indicated that VW5 could penetrate the bacterial membrane in a similar manner as melittin and exhibited stronger outer membrane permeability and cytoplasmic membrane depolarization ability. It has been long reported that the net positive charge of peptides was of vital importance in the antimicrobial performance (Jiang et al., 2008). Furthermore, it was confirmed that the morphological change of bacteria to the peptides could cause the disruption of the bacterial membrane and leakage of the cytoplasmic content (Li et al., 2021; Wang et al., 2021).

A bacterial infection will induce the neutrophils invading into the abscess site through blood vessels at the early phase of abscess, and the aggregated neutrophils could capture bacteria and then secrete various kinds of proteases and reactive oxygen metabolites to ingest bacteria (Graeme and Guido, 1977; Trowbridge, 2010). However, neutrophils autolysis also inevitably occurred which was induced by their own lysosomal enzymes during the process of bacterial ingestion, and the proteases and reactive oxygen metabolites were released into the surrounding environment inducing normal tissue destruction (Kolar and Liu, 2016). Eventually, the abscess was formed, which was composed of various complicated elements such as pus, a large amount of cell debris, and bacteria (Hays and Mandell, 1974), which would invalidate the efficacy and penetration of most antibiotics. In this study, the bacterial load at the abscess lesions and the area of necrotic tissue significantly decreased, which indicated that the peptide VW5 was equipped with excellent *in vivo* antimicrobial activity. The proinflammatory cytokines, produced by active macrophages, has long been regarded as the indicator of the degree of inflammation because of the ability to upregulate the inflammation response (Müller et al., 2017). The level of cytokines in serum was significantly reduced after VW5 treatment, which signifies an excellent therapeutic effect for bacterial infection. The result of histological assay, in which the skin structure was relatively intact after VW5 treatment compared to control, further reflected the therapeutic potential of VW5. Through the DAPI assays, the antimicrobial peptide VW5 did not exhibit green fluorescence, indicating that the antimicrobial peptide did not cause cell apoptosis and was equipped with skin toxicity.

## Data Availability Statement

The original contributions presented in the study are included in the article/supplementary material, further inquiries can be directed to the corresponding author.

## Ethics Statement

The studies involving human participants were reviewed and approved by the ethics committee of Laboratory Animals of the Northeast Agricultural University. The patients/participants provided their written, informed consent to participate in this study. The animal study was reviewed and approved by the Care and Use of Laboratory Animals of the Northeast Agricultural University (protocol code, NEAU-[2011]-9).

## Author Contributions

ZW was responsible for the experimental design, experimental implementation, and data analysis of the whole experiment. QL performed *in vitro* antibacterial experiments. JL oversaw the implementation of the mouse experiment. JL performed SEM and TEM assays. LS operated HE and DAPI staining. SC carried out the colony counting and analysis. YL analyzed the slice of samples. AS was in charge of supervision and article reviewing. All authors contributed to the article and approved the submitted version.

## Funding

We gratefully acknowledge the financial support from the National Natural Science Foundation of China [32030101, 31872368, 31672434], the China Agriculture Research System of MOF and MARA, and the Natural Science Foundation of Heilongjiang Province [TD2019C001].

## Conflict of Interest

The authors declare that the research was conducted in the absence of any commercial or financial relationships that could be construed as a potential conflict of interest.

## Publisher’s Note

All claims expressed in this article are solely those of the authors and do not necessarily represent those of their affiliated organizations, or those of the publisher, the editors and the reviewers. Any product that may be evaluated in this article, or claim that may be made by its manufacturer, is not guaranteed or endorsed by the publisher.
